# Artificial intelligence readiness in fragile states: a systematic review and theory-informed framework for workforce transformation in Somalia

**DOI:** 10.3389/frai.2026.1928521

**Published:** 2026-10-16

**Authors:** Ayan Mohamed Jama, Nashad Farah Mohamud, Mohamud Osman Hamud, Abdullahi Mohamud Hassan, Abukar Sheikh Mohamed

**Affiliations:** 1 Faculty of Computer Science, Somali National University, Mogadishu, Somalia; 2 Somali AI Network, Mogadishu, Somalia; 3 Dugsiiye, Mogadishu, Somalia

**Keywords:** AI readiness, digital skills gap, fragile states, human capital, Somalia, technology-organization-environment framework, workforce productivity

## Abstract

**Background:**

Artificial intelligence (AI) is increasingly recognized as a determinant of national productivity and competitiveness, yet the conditions required for its beneficial adoption, infrastructure, institutions, and human capital, remain unevenly distributed across the globe. Somalia, a fragile and conflict-affected state undergoing a prolonged process of institutional reconstruction, exemplifies this unevenness: a young, digitally engaged population and a strikingly dynamic private telecommunications and mobile-money sector coexist with weak public institutions, fragmented higher education, and chronic graduate unemployment.

**Methods:**

This paper reports a systematic literature review with theory-informed framework development, conducted in accordance with the PRISMA 2020 reporting guideline, that synthesizes peer-reviewed evidence on organizational and national AI readiness, digital-skills development, and workforce productivity, with a specific focus on Somalia and comparable fragile, low-connectivity, Sub-Saharan African contexts. Using the Technology–Organization–Environment (TOE) framework and human capital theory as complementary lenses, the review synthesizes findings from 31 sources identified through a structured search and screening process.

**Results:**

The evidence indicates that Somalia’s AI readiness gap is not primarily a technological deficit but a multidimensional one, spanning fragmented digital infrastructure, weak institutional governance and data ecosystems, a persistent mismatch between higher-education curricula and labour-market needs, and the absence of coordinated national AI or digital-skills policy.

**Conclusion:**

Building on these findings, the paper proposes a four-pillar conceptual AI readiness framework intended to guide future empirical assessment and policy discussion, encompassing digital infrastructure and access, institutional and governance readiness, human capital and skills development, and labour-market integration, together with a phased implementation roadmap for government, higher-education institutions, the private telecommunications sector, and development partners. The review provides a literature-grounded, context-specific synthesis of AI readiness for a fragile Horn of Africa state and offers a transferable analytical approach for similar settings.

## 1 Introduction

Artificial intelligence is widely portrayed in the economics and management literature as a general-purpose technology capable of reshaping labour productivity, organizational decision-making, and national competitiveness. Firm-level and cross-industry evidence links AI adoption to measurable productivity gains, particularly where AI augments rather than merely displaces skilled labour, and where the workforce possesses the complementary competencies required to exploit new tools effectively (Hirtranusi et al., 2026). Over 60 countries have published national AI strategies; more than 70% of these frameworks belong to developed nations (Demaidi, 2023). At the same time, a substantial body of research shows that the benefits of AI are conditional on “readiness,” a multidimensional state encompassing technological infrastructure, organizational and institutional capability, and a supportive external environment (Hirtranusi et al., 2026). Where these preconditions are absent, AI adoption stalls, is captured by a narrow elite, or fails to translate into broad-based productivity gains.

For Sub-Saharan Africa, and for fragile and conflict-affected states in particular, this readiness gap is especially pronounced. Reviews of AI adoption on the continent consistently identify inadequate infrastructure, scarce data ecosystems, weak regulatory frameworks, and, above all, shortages of relevantly skilled personnel as the principal barriers to translating AI’s potential into development outcomes (Mienye et al., 2024; Aderibigbe et al., 2023). Somalia occupies a distinctive position within this regional picture. Three decades of state fragmentation and conflict produced one of the weakest formal-institution environments in the world, yet the same period saw an unusually vigorous, privately led expansion of mobile telecommunications and mobile money, to the point that Somalia has developed a relatively advanced privately led mobile-money ecosystem compared with many fragile-state contexts in Sub-Saharan Africa (Chonka et al., 2025). This juxtaposition, strong grassroots digital uptake against weak formal institutions and human-capital systems, makes Somalia an instructive, under-examined case for understanding what “AI readiness” means in a fragile-state context, and what a realistic evidence-informed pathway toward it would require.

Somalia’s higher-education sector has expanded rapidly in the past decade, yet this expansion has not translated into an employable, technologically capable workforce. The country ranks at the bottom of global AI readiness indices, with critical gaps in digital infrastructure, limited access to quality education, and weak institutional frameworks. Decades of instability have left little room for advancements in digital infrastructure or education. The Global Index on Responsible AI reports that Somalia received scores of 0.26 for Government Frameworks (ranking 137th), 0.26 for Government Actions (ranking 137th), and 0.41 for Non-State Actors (ranking 115th) (Adams et al., 2024). National assessments report that a majority of new graduates lack essential employability skills, including digital literacy, and that skills mismatch between what universities teach and what employers need is a leading driver of graduate unemployment, alongside weak career guidance, outdated technical and vocational curricula, and non-meritocratic hiring practices (Ahmed, 2023; Yasin Gelle et al., 2021). At the same time, empirical assessments of Somali public institutions find that although government employees are relatively young and express strong motivation to build digital competence, actual ICT training provision is *ad hoc*, unevenly distributed across ministries, and rarely embedded in a coordinated capacity-building strategy. Somalia has, to date, no dedicated national AI strategy or AI-specific readiness assessment comparable to those beginning to appear elsewhere on the continent. This leaves policymakers, universities, and development partners without a literature-grounded framework for sequencing investment in digital infrastructure, institutional governance, human capital, and labour-market integration. The problem this review addresses, therefore, is twofold: first, to establish what the existing peer-reviewed evidence base indicates about the components and determinants of AI readiness in fragile, low-connectivity, human-capital-constrained settings; and second, to translate that evidence into a proposed conceptual framework tailored to Somalia’s specific institutional and labour-market conditions.

The skilled workforce sectors most immediately relevant to AI readiness in Somalia include telecommunications and financial services (where infrastructure already exists), public administration (where governance capacity must be built), health and education (where service delivery could benefit from AI augmentation), and agriculture (the largest employment sector, where AI could improve productivity and resilience). However, the evidence base on Somalia’s digital and AI readiness is concentrated in the telecommunications, education, and graduate-employment sectors, with scarce peer-reviewed research on AI-specific readiness in health, agriculture, or public administration. This uneven evidence base shapes the review’s focus and is itself an important finding of the synthesis.

This review pursues three objectives: (1) to systematically identify and synthesize peer-reviewed literature on AI readiness, digital-skills development, and workforce productivity as they apply to Somalia and comparable fragile, Sub-Saharan African contexts; (2) to characterize, through that synthesis, the principal dimensions of Somalia’s AI readiness gap; and (3) to derive, from the synthesized evidence, a proposed conceptual framework for enhancing skilled workforce productivity through improved AI readiness.

Somalia remains almost absent from the rapidly growing literature on national and organizational AI readiness, which is dominated by studies of higher-income and larger middle-income economies. By systematically consolidating the fragmented but genuine body of peer-reviewed research on Somali ICT readiness, digital skills, and labour-market dynamics, and by situating it within established AI readiness and human-capital theory, this review offers three contributions. First, it provides scholars with a consolidated evidence base and identifies concrete gaps for future empirical research in fragile-state contexts. Second, it offers Somali policymakers, universities, and the private telecommunications sector an evidence-grounded framework rather than an imported, decontextualized readiness checklist. Third, it contributes a transferable analytical approach, combining the TOE framework with human capital theory, that other fragile and conflict-affected states may adapt.

## 2 Theoretical and conceptual framework

AI readiness has been defined inconsistently across the literature, with prior studies varying in their treatment of definitions, constituent factors, and analytical approaches (Hirtranusi et al., 2026). A recent PRISMA-guided systematic review consolidated 124 readiness-related indicators drawn from the peer-reviewed literature into 35 factors and, ultimately, 12 core readiness themes, concluding that AI readiness is best understood not as a single condition but as a multidimensional, interdependent construct shaped by the alignment of technological capability, organizational structure and competence, and external environmental conditions (Hirtranusi et al., 2026). This conceptualization cautions against treating AI readiness as synonymous with infrastructure or connectivity alone, a simplification that would be particularly misleading in the Somali context, where telecommunications infrastructure is comparatively advanced relative to institutional and human-capital readiness.

This review adopts the Technology-Organization-Environment (TOE) framework as its primary analytical lens, complemented by human capital theory to explain the productivity mechanism. The TOE framework, which explains technology adoption as a function of technological, organizational, and environmental factors, has become the dominant analytical lens in the organizational AI readiness literature and has been explicitly applied to characterize AI readiness across African countries (Hirtranusi et al., 2026). Its explanatory power lies in disaggregating readiness into technological factors (infrastructure, data availability, system compatibility), organizational factors (management support, structure, absorptive capacity, workforce competence), and environmental factors (regulatory frameworks, competitive pressure, institutional and donor support). This review adopts the TOE framework as its primary organizing lens for synthesizing findings, while recognizing that readiness should be treated as a systemic, evolving capability rather than a static checklist (Hirtranusi et al., 2026).

Human capital theory, originating from foundational analysis of investment in skills and education as a determinant of individual and aggregate productivity, provides the second theoretical pillar (Zhai et al., 2025). Firm-level evidence indicates that AI technology raises total factor productivity substantially by increasing the proportion of highly skilled labour within firms and by generating capital-skill complementarities (Fan et al., 2025; Gao and Feng, 2023), implying that organizations or economies with weaker skill bases capture a smaller share of AI’s potential productivity gains even where the technology itself is accessible. AI readiness should not be conceptualized as a direct determinant of workforce productivity but rather as an enabling condition that facilitates successful AI adoption and utilization. The productivity effects of AI emerge through intermediate mechanisms, including organizational absorptive capacity, workforce adaptability, managerial support, sector-specific implementation strategies, and effective integration of AI tools into existing workflows. Therefore, this review conceptualizes the relationship between AI readiness and productivity as a sequential pathway in which technological, organizational, and environmental readiness strengthen AI adoption capability, which subsequently enables workforce augmentation and productivity improvement.

The Unified Theory of Acceptance and Use of Technology (UTAUT) further explains individual-level technology adoption as determined by performance expectancy, effort expectancy, social influence, and facilitating conditions (Venkatesh et al., 2003). Fragile and conflict-affected states are frequently excluded from mainstream technology-adoption research, yet technical solutions succeed only when paired with sustained institutional capacity-building, coordinated donor engagement, and context-specific design (El-Jardali et al., 2023). Somalia’s experience with mobile money illustrates this dynamic vividly: an indigenous, privately led digital-finance ecosystem emerged precisely because formal state capacity was absent, with telecommunications conglomerates assuming quasi-state functions in payments, connectivity, and, increasingly, humanitarian cash-transfer infrastructure (Chonka et al., 2025). This suggests that any Somalia-specific AI readiness framework must account for a private sector that is simultaneously a source of digital capability and a potential obstacle to coordinated, state-led digital governance.

### 2.1 Conceptual pathway linking AI readiness to workforce productivity

Based on the TOE framework and human capital theory, this review proposes that AI readiness influences workforce productivity through a mediated pathway rather than a simple direct relationship. Technological readiness provides access to digital infrastructure, data resources, and AI-enabled tools. However, these technological assets generate productivity benefits only when supported by organizational readiness, including institutional capacity, leadership commitment, governance mechanisms, and workforce capability. Human capital readiness determines whether individuals and organizations can effectively utilize AI technologies for decision-making, innovation, and task augmentation. Thus, the proposed pathway is presented in Figure 1 below.

**Figure 1:**
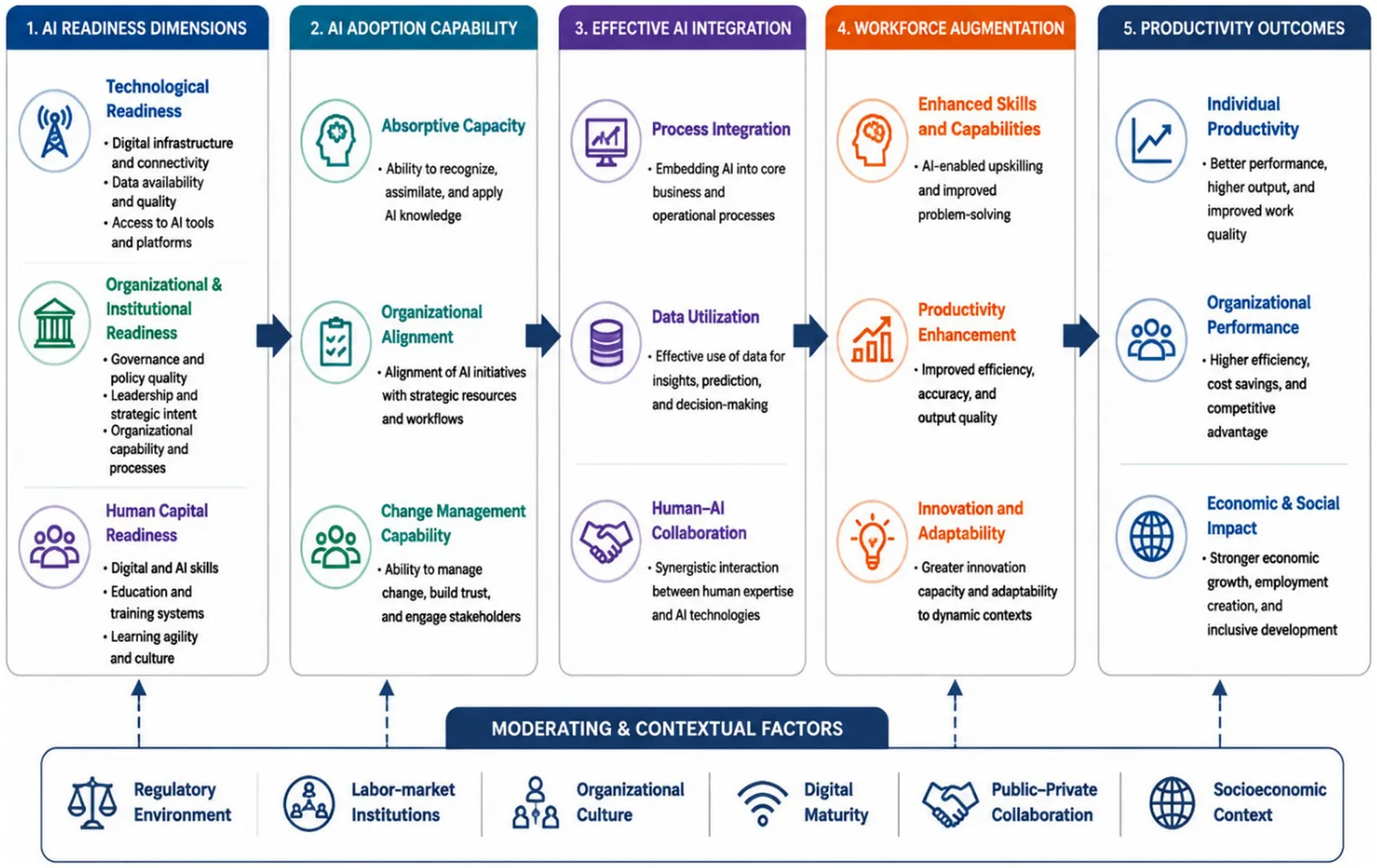
Conceptual pathway linking AI readiness dimensions, AI adoption capability, and workforce productivity.

### 2.2 Research propositions

Based on the theoretical framework, this review advances the following propositions:

Proposition 1: Technological readiness alone is insufficient to generate productivity benefits unless supported by organizational and institutional readiness.

Proposition 2: Human capital readiness moderates the relationship between AI adoption capability and workforce productivity by determining the ability of workers to effectively utilize AI technologies.

Proposition 3: Labour-market integration mechanisms influence whether AI-related skills translate into employment opportunities and productivity gains.

Proposition 4: In fragile states, private-sector digital infrastructure can accelerate AI readiness but requires complementary public governance mechanisms to ensure inclusive and sustainable adoption.

## 3 Methodology

### 3.1 Review design and reporting standard

This study was conducted as a systematic literature review and reported in accordance with the Preferred Reporting Items for Systematic Reviews and Meta-Analyses (PRISMA) 2020 statement (Page et al., 2021). The PRISMA 2020 guideline was selected because it provides a transparent, replicable framework for reporting the identification, screening, eligibility assessment, and inclusion of studies, and is now the expected reporting standard across management, information systems, education, and health-policy journals in which AI readiness scholarship is published. A systematic review methodology was chosen to ensure a comprehensive, unbiased synthesis of the fragmented evidence on AI readiness, digital skills, and workforce productivity as they apply to Somalia and comparable fragile-state contexts. Given the heterogeneity of the included literature and the absence of quantitative data suitable for meta-analysis, a narrative synthesis approach was adopted, consistent with guidance for reviews in emerging and interdisciplinary fields where study designs and outcome measures vary considerably.

### 3.2 Grey literature exclusion rationale

Although grey literature sources such as national digital strategies, policy documents, international development reports, and AI readiness indices are highly relevant for policy-oriented research, they were excluded from the formal evidence synthesis because the primary objective of this review was to establish a transparent and reproducible evidence base derived from peer-reviewed scholarship. However, selected high-relevance contextual reports and indices were considered separately to support background interpretation and contextual benchmarking. These sources were not weighted as equivalent to peer-reviewed evidence in the thematic synthesis.

### 3.3 Search strategy

A structured search was conducted in July 2026 across multiple academic databases and repositories. Searches were performed across the following databases: Scopus, Web of Science Core Collection, Google Scholar, PubMed, and ERIC (Education Resources Information Center). Supplementary searches were conducted in publisher-specific platforms including ScienceDirect, Taylor & Francis Online, SpringerLink, Oxford Academic, BMJ Journals, and Frontiers. The search strategy combined three thematic categories of terms using Boolean operators, with the core search string structured as follows: (“AI readiness” OR “artificial intelligence readiness” OR “digital readiness” OR “technology readiness” OR “ICT readiness”) AND (“Somalia” OR “fragile state*” OR “conflict-affected” OR “post-conflict” OR “Sub-Saharan Africa” OR “East Africa”) AND (“workforce” OR “productivity” OR “human capital” OR “skill*” OR “employment” OR “labour market” OR “employment”) AND (“adoption” OR “implementation” OR “integration” OR “diffusion” OR “capacity building”). Twenty distinct search queries were executed across successive rounds, yielding a pool of candidate records that were then screened for peer-review status and topical relevance. All searches were conducted in English. Reference lists of included studies were hand-searched to identify additional relevant sources not captured by database searches (forward and backward citation tracking).

### 3.4 Study selection process

Following the completion of the database searches, all identified records were exported to reference management software where duplicates were identified and removed. The study selection process was conducted in two stages. (Stage 1) Title and Abstract Screening. Two reviewers independently screened the titles and abstracts of all identified records against the eligibility criteria. Records that clearly did not meet the inclusion criteria were excluded. Disagreements between reviewers were resolved through discussion, with a third reviewer consulted where consensus could not be reached. (Stage 2) Full-Text Review. Full texts of all potentially eligible records were retrieved and assessed against the eligibility criteria. Reasons for exclusion at this stage were documented. The final set of included studies was determined through consensus among all authors. Figure 2 summarizes the flow of records through the review, adapted from the PRISMA 2020 flow diagram (Page et al., 2021).

**Figure 2:**
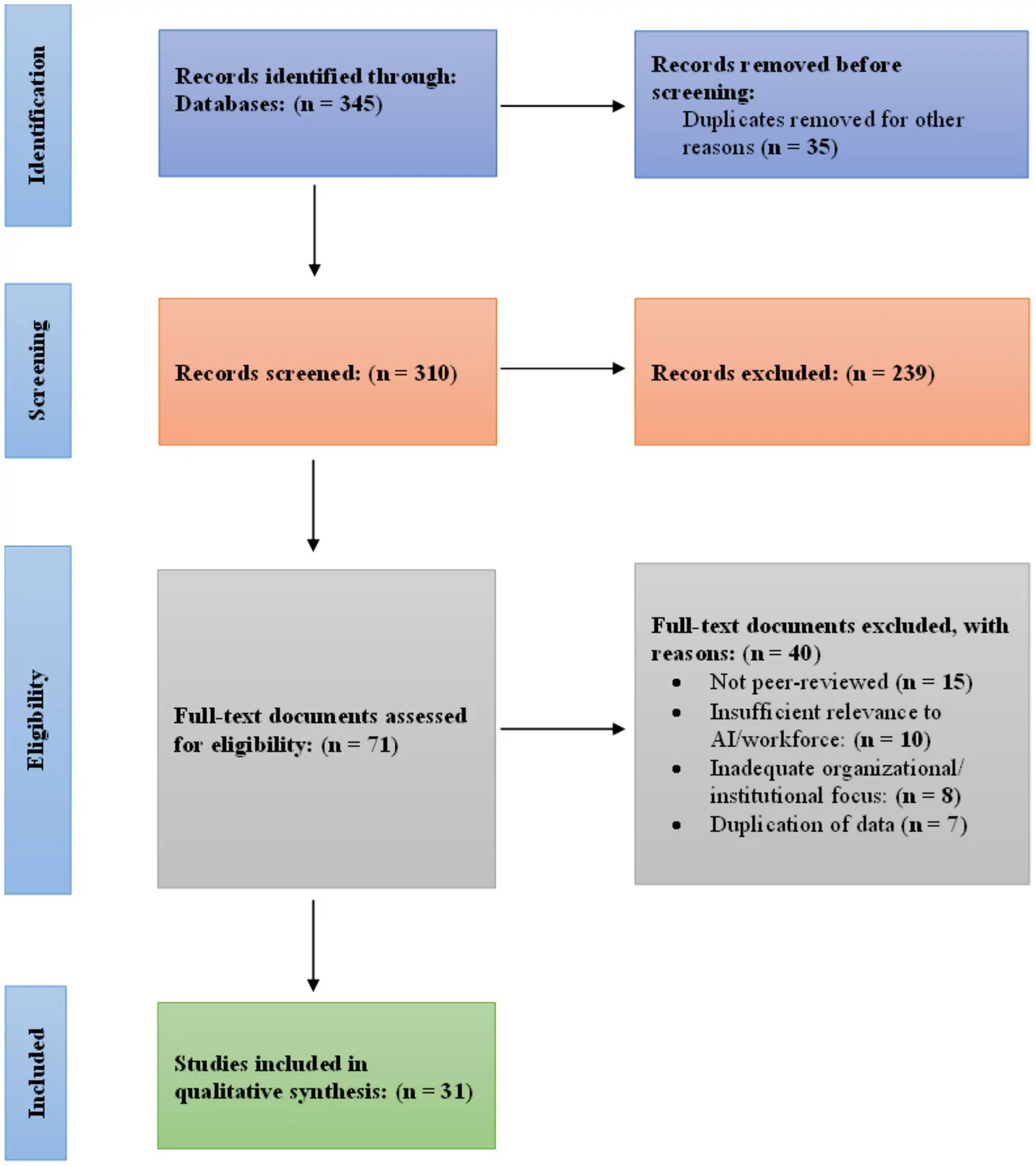
PRISMA 2020 flow diagram of the study selection process.

### 3.5 Quality assessment

The methodological quality of included studies was assessed using design-appropriate critical appraisal tools. The Joanna Briggs Institute (JBI) critical appraisal checklists were used for empirical and review studies, the Critical Appraisal Skills Programme (CASP) checklists were applied to qualitative studies, and AMSTAR-2 was used for systematic reviews and meta-analyses. Because grey literature sources were not included as equivalent evidence within the formal peer-reviewed synthesis, AACODS appraisal was only applied to selected contextual sources used for background interpretation. Quality appraisal was conducted independently by two reviewers. Each study was assessed according to the relevant appraisal criteria for its methodological design, considering aspects such as study design appropriateness, methodological transparency, data collection and analysis procedures, validity of findings, and alignment with the review objectives. Any disagreements between reviewers were resolved through discussion and consensus. Based on the proportion and relevance of appraisal criteria fulfilled within each assessment instrument, studies were categorized as having high, moderate, or low confidence. Given the heterogeneity of included study designs and appraisal instruments, results were interpreted comparatively rather than as a pooled numerical quality score. Quality ratings were not used as an exclusion criterion; instead, they informed the interpretation and weighting of evidence during thematic synthesis and framework development. The detailed quality appraisal results are presented in Supplementary Table 1.

### 3.6 Data extraction and synthesis

For each included study, the following data were extracted using a standardized extraction form: authorship and year of publication; geographic and sectoral scope; theoretical framework applied; methodology (study design, data collection methods, and analytical approach); principal findings relevant to AI readiness, digital skills, or workforce productivity; and policy or strategic recommendations (Supplementary Table 2). Data extraction was initially performed independently by two reviewers using a standardized extraction template. Extracted information was compared for consistency, and discrepancies were resolved through discussion. For records where differences remained, a senior reviewer adjudicated the final decision. Extracted domains included study characteristics, methodological approach, theoretical framework, AI readiness dimensions, digital-skills findings, workforce implications, and policy recommendations.

Thematic synthesis involved: (a) coding of findings from each included study according to the relevant TOE dimension and thematic category; (b) grouping of codes into broader themes; and (c) narrative description of each theme with illustrative evidence from the included sources. Coding followed both deductive and inductive approaches. Deductive coding was guided by TOE dimensions (technological, organizational, and environmental factors), while inductive coding allowed additional themes to emerge from the included studies. Two reviewers independently reviewed thematic categories and resolved discrepancies through discussion and consensus. Themes were retained when supported by multiple studies and demonstrated relevance to the Somali context. Cross-cutting themes and interrelationships between dimensions were explored iteratively, with particular attention to feedback loops in which a barrier in one dimension was found to compound barriers elsewhere in the system.

### 3.7 Limitations of the study

Three methodological limitations should be noted transparently. First, all database searches were conducted using direct database interfaces, including Scopus, Web of Science Core Collection, PubMed, ERIC, and Google Scholar. The complete search strings, database-specific syntax, search dates, filters, and retrieved records are provided in Supplementary Appendix 1 to ensure reproducibility. Second, the peer-reviewed literature specifically addressing Somalia is genuinely thin, a finding that is itself substantively important, but which necessarily limits the granularity of Somalia-specific synthesis relative to the broader Sub-Saharan African and global evidence used to contextualize it. Third, because eligibility was restricted to peer-reviewed sources, several recent and topically relevant preprints and policy reports (including 2025–2026 AI readiness indices for Africa) were deliberately excluded despite their relevance, which may omit very recent developments not yet reflected in peer-reviewed outlets.

The exclusion of grey literature represents a deliberate methodological boundary rather than an assumption that non-peer-reviewed evidence lacks value. Additionally, restricting inclusion to English-language peer-reviewed publications may have introduced publication and language bias, particularly in contexts where digital transformation evidence is often documented through institutional reports. In fragile and conflict-affected settings such as Somalia, government strategies, donor assessments, and implementation reports may contain important operational insights that are not captured in academic databases. However, including such sources would have introduced additional methodological variability requiring separate appraisal procedures. Therefore, this review prioritized methodological consistency by limiting formal synthesis to peer-reviewed evidence. Future research should complement this evidence base through a dedicated mixed evidence review incorporating grey literature assessed using AACODS or equivalent appraisal frameworks.

### 3.8 Strengths of the review

This review has several strengths. First, it applies a theoretically grounded framework combining TOE and human capital theory to examine AI readiness in a fragile-state context. Second, it synthesizes evidence from Somalia and comparable African settings to generate context-specific insights. Third, the PRISMA-guided methodology enhances transparency and reproducibility.

## 4 Results

Thirty-one peer-reviewed sources met the eligibility criteria and were retained for synthesis (Supplementary Table 2), using six thematic domains: (a) Technological Readiness, (b) Institutional and Governance Readiness, (c) Human Capital, Digital Skills, and Higher Education, (d) AI Adoption, Human Capital, and Productivity, (e) Financial and Mobile-Money Infrastructure as a Productivity Channel, and (f) Comparative Digital Capacity-Building Evidence from Analogous African Sectors.

### 4.1 Technological readiness

Across the AI readiness literature, technological factors, infrastructure, data ecosystems, and system compatibility are consistently identified as necessary but insufficient conditions for readiness (Hirtranusi et al., 2026). At the continental level, reviews of AI adoption in Africa identify inadequate infrastructure and limited access to technology, particularly in rural areas, as persistent constraints, compounded by scarce structured data ecosystems that limit the training and deployment of context-relevant AI applications (Mienye et al., 2024; Aderibigbe et al., 2023). Somalia’s position within this technological picture is genuinely distinctive: rapid, privately financed expansion of mobile telecommunications and fibre-optic connectivity, combined with an indigenous mobile-money ecosystem that now underpins a large share of everyday commerce, has produced a level of last-mile digital access that outpaces many peers’ fragile states (Chonka et al., 2025). However, this infrastructure was built to serve payments and voice/data communication, not a coordinated national data or AI ecosystem, and its governance sits substantially outside formal state institutions, a structural feature with direct implications for any future AI readiness strategy (Chonka et al., 2025).

### 4.2 Institutional and governance readiness

The organizational and environmental dimensions of the TOE framework, management support, regulatory frameworks, and institutional capacity, emerge across the literature as the areas of greatest weakness in African and fragile-state contexts. Continental reviews point to insufficient government policy frameworks and regulatory uncertainty as key barriers to AI adoption (Aderibigbe et al., 2023), while research specifically on Africa’s AI positioning argues that countries in the Global South must first address governance deficits and institutional capacity gaps before AI can be expected to flourish, and highlights the role international partners could play in bridging these gaps through a problem-driven, rather than technology-first, approach (Arakpogun et al., 2021). Somalia’s case sharpens this general finding: because formal state capacity in payments, connectivity, and data governance has historically been so limited, the commercial telecommunications sector has assumed quasi-sovereign functions across financial infrastructure and, increasingly, humanitarian and social-protection payment systems (Chonka et al., 2025). This concentration of digital governance capacity within a small number of private conglomerates, rather than public institutions, means that institutional AI readiness in Somalia cannot be pursued through conventional government-led digital-strategy processes alone; it requires explicit engagement with, and possibly formal partnership arrangements involving, the private telecommunications sector.

### 4.3 Human capital, digital skills, and higher education

Human capital constraints appear as the most frequently cited barrier to AI adoption across the reviewed literature, and the evidence on Somalia’s education-to-employment pipeline substantiates this concern in granular detail. A systematic review of digital literacy in Sub-Saharan African higher-education institutions found that 21st-century digital skills remain insufficiently developed across the region because of inadequate digital infrastructure in universities, underprepared lecturers, and outdated or absent institutional ICT policy, and called for curricular reform prioritizing digital solutions and continuous professional development for educators (Ndibalema, 2025). A complementary systematic review of 21st-century skills education across Sub-Saharan Africa similarly found that life-skills and soft-skills integration into education systems remains inconsistent, constrained by infrastructural gaps, teacher preparedness, and the absence of culturally adapted, validated assessment tools (Akongo et al., 2025). Technical and vocational education, the pathway most directly tied to workforce productivity, shows a parallel pattern: a literature review focused on Ethiopia found that weak linkage between vocational training institutions and industry is a central, remediable cause of skills mismatch, and that structured mechanisms such as qualification standard-setting, curriculum co-development, and internships can substantially improve the transition from training to productive employment (Kebede et al., 2024).

A separate scoping review across Nigeria, South Africa, and the wider Sub-Saharan region likewise found that digital-skills development measurably enhances youth employability and that digital platforms independently foster entrepreneurship by expanding access to markets and self-employment opportunities, even where formal employment remains scarce (Neema Mariette et al., 2025). Within Somalia specifically, a secondary-data review of higher-education digital transformation found that although national ICT-in-education policy processes are underway, digital learning readiness remains highly uneven across institutions and regions, shaped by disparities in connectivity, affordable data, computing devices, and institutional support (Hassan et al., 2026). Empirical labour-market research reinforces this picture from the demand side: Somali graduates are consistently reported as strong in theoretical knowledge but weak in the practical, digital, and problem-solving competencies employers require, with skills mismatch identified as a leading structural driver of graduate unemployment, compounded by weak career guidance, outdated technical and vocational curricula, and non-meritocratic recruitment practices that further depress the return to skill (Ahmed, 2023; Yasin Gelle et al., 2021).

Across the reviewed studies, there is strong convergence regarding the importance of human capital, governance, and organizational capability as determinants of AI readiness. However, the literature differs in explaining the origin of readiness gaps. Studies focused on higher education emphasize curriculum limitations and instructor capacity, whereas labour-market studies highlight employer expectations, recruitment systems, and weak university–industry connections. These perspectives suggest that AI readiness gaps are produced through interacting educational, institutional, and economic factors rather than a single constraint.

### 4.4 AI adoption, human capital, and productivity

At the level of underlying economic mechanisms, the evidence base establishes that AI’s productivity dividend is mediated by, rather than independent of, workforce human capital, consistent with Becker’s (1962) original account of human capital as a productivity-augmenting investment. Firm-level research demonstrates that AI technology raises total factor productivity substantially by increasing the share of highly skilled labour within adopting firms and by generating capital-skill complementarity (Fan et al., 2025; Gao and Feng, 2023), implying that organizations or economies with weaker skill bases capture a smaller share of AI’s potential productivity gains even where the technology itself is accessible.

Complementary evidence on employment structures shows that AI and technological innovation reshape who benefits from the digital economy, with outcomes depending heavily on pre-existing digital and human-capital readiness rather than on technology access alone (Uddin et al., 2026), while broader sector-agnostic surveys of AI adoption in small and medium-sized enterprises identify organizational readiness, digital infrastructure, and human capital jointly, rather than any single factor, as the decisive conditions separating firms that capture AI’s benefits from those that remain at an exploratory stage (Yesuf and Fields, 2025). This mechanism is directly relevant to Somalia, because the country’s technological readiness (connectivity, mobile-money infrastructure) currently exceeds its human-capital readiness; the marginal productivity return to further infrastructure investment is likely to be constrained unless matched by parallel investment in digital and AI-relevant skills.

### 4.5 Financial and Mobile-money infrastructure as a Productivity Channel

A distinct but related strand of evidence documents how mobile financial infrastructure, of the kind Somalia has built extensively, can itself function as a productivity- and welfare-enhancing channel when paired with adequate digital and financial literacy. The seminal study of Kenya’s M-PESA system found that reduced transaction costs from mobile money materially improved households’ ability to share risk and smooth consumption after economic shocks, establishing mobile money as a genuine driver of household-level economic resilience in East Africa (Jack and Suri, 2014). Continent-wide evidence extends this finding, showing that digital financial inclusion in Africa is shaped jointly by infrastructure access and by digital literacy, with women, less-educated, and lower-income populations facing disproportionate exclusion even where mobile-money infrastructure itself is widely available (Elouaourti and Ibourk, 2024).

Complementary cross-regional evidence spanning Africa, Asia, and Latin America confirms that digital literacy and technology adoption jointly, rather than infrastructure access alone, determine the extent to which digital financial services translate into genuine financial inclusion (Adel, 2024), while sub-Saharan Africa-specific evidence links digital financial services adoption directly to measurable gains in economic inclusion (Osuma, 2025). For Somalia, where mobile money already underpins a large share of household and small-enterprise transactions, this literature suggests that the existing financial-technology infrastructure is a genuine national asset, but that its full productivity potential will remain unrealized without parallel, deliberate investment in digital and financial literacy across the population.

### 4.6 Comparative digital capacity-building evidence from analogous African sectors

Because peer-reviewed, Somalia-specific AI readiness data remain scarce, this review also draws on closely analogous evidence from digital capacity-building in African health and public-institution settings, which share Somalia’s core structural constraints of infrastructure gaps, workforce shortages, and fragmented governance. Assessments of digital competency among health-profession educators in Ethiopia found that only a small minority reached advanced digital-skill levels, with the majority clustered at beginner or exploratory stages despite operating within functioning public institutions, underscoring how substantial the digital-skills gap can be even outside conflict-affected settings (Amare et al., 2024). A systematic review and meta-analysis of digital literacy among Ethiopian health professionals similarly found highly variable digital-literacy levels strongly associated with prior training exposure and institutional support (Chereka et al., 2024), while a multidimensional capacity assessment across three African health institutions found that data governance, infrastructure, and workforce training capacity varied so substantially between otherwise comparable institutions that a uniform, one-size-fits-all capacity-building approach was explicitly rejected in favor of institution-specific roadmaps (Kiragga et al., 2025).

Evidence from the clinical AI readiness literature reinforces this institutional lesson: a systematic review of nurses’ and nursing students’ AI readiness found that attitudes and self-reported readiness consistently outpaced actual AI literacy, with UTAUT-consistent factors such as facilitating conditions and organizational support determining whether positive attitudes translated into effective use (El Arab et al., 2025). Taken together, this cross-sectoral evidence reinforces the review’s central finding regarding Somalia: positive intent and even comparatively strong underlying infrastructure are not, by themselves, sufficient for readiness; structured, sustained, institutionally embedded training is the binding constraint.

### 4.7 Synthesis against the TOE framework

Table 1 maps the principal findings from the included studies onto the three TOE dimensions, together with an assessment of Somalia’s relative position on each, derived from the Somalia-specific and comparative Sub-Saharan African evidence reviewed above.

**Table 1:** Synthesis of AI readiness evidence using the TOE framework and human capital perspective, with Somalia-specific evidence interpretation.

TOE dimension	General sub-Saharan African/fragile-state evidence	Somalia-specific evidence interpretation
Technological	Infrastructure and structured data ecosystems widely inadequate; rural access especially limited (Mienye et al., 2024; Aderibigbe et al., 2023)	Comparatively strong mobile/telecom and mobile-money infrastructure, privately built; weak public data ecosystem and no coordinated AI/data infrastructure (Chonka et al., 2025)
Organizational	Weak regulatory frameworks and limited government AI policy across much of the continent (Aderibigbe et al., 2023; Arakpogun et al., 2021)	No national AI strategy; digital governance capacity concentrated in private telecom conglomerates rather than public institutions (Chonka et al., 2025); uneven, uncoordinated ICT training in government institutions
Human Capital	Digital-literacy and 21st-century-skills gaps widespread in higher education; teacher/lecturer preparedness and curricula lag technology (Chonka et al., 2025; Ndibalema, 2025)	Uneven digital-learning readiness across universities; graduate skills mismatch and weak employability skills a leading driver of unemployment (Ahmed, 2023; Yasin Gelle et al., 2021; Hassan et al., 2026)

## 5 Discussion

The synthesized evidence supports a central, non-obvious conclusion: Somalia’s AI readiness gap is not primarily a connectivity or access problem. Relative to many low-income and fragile peers, Somalia’s telecommunications and mobile-money infrastructure is comparatively advanced (Chonka et al., 2025). The binding constraints instead sit in the organizational/institutional and human-capital dimensions of the TOE framework: the absence of coordinated public-sector digital and data governance, and a chronic, well-documented mismatch between what higher education produces and what a digitally augmented labour market requires (Ahmed, 2023; Yasin Gelle et al., 2021). This inverts the assumption, implicit in many generic AI readiness checklists, that infrastructure is the foundational layer on which institutional and human-capital readiness are subsequently built. In Somalia, infrastructure arrived first, largely through private initiative, while institutional and human-capital readiness lag substantially behind.

The transferability of international evidence requires careful interpretation. Evidence from advanced economies, including firm-level studies from China and other high-income settings, primarily explains productivity mechanisms after AI adoption has already occurred. In contrast, Somalia remains at an earlier readiness stage characterized by institutional constraints and limited AI deployment. Therefore, evidence from developed economies is used primarily to explain theoretical mechanisms, whereas evidence from fragile states and Sub-Saharan African contexts receives greater contextual relevance for policy adaptation.

Somalia’s pattern both confirms and complicates the broader regional picture. It confirms the region-wide finding that skills acquisition and institutional/regulatory weakness, more than raw technology access, are the dominant barriers to beneficial AI adoption (Mienye et al., 2024; Aderibigbe et al., 2023). It complicates the regional picture, however, insofar as most African AI readiness literature assumes a still-functioning, if resource-constrained, central state as the primary readiness-building actor (Arakpogun et al., 2021). Somalia’s experience shows that in a fragile state where private actors have already built substantial digital infrastructure and assumed quasi-governmental functions, an AI readiness strategy must be co-designed with those private actors rather than assuming state primacy, a nuance largely absent from generic continental frameworks.

Although AI presents significant opportunities for improving productivity, service delivery, and innovation, adoption in fragile states may also generate unintended consequences. Potential risks include automation-related displacement of routine occupations, widening inequality between digitally skilled and digitally excluded populations, dependence on external technology providers, and reinforcement of existing institutional weaknesses where governance capacity remains limited. In Somalia, where digital infrastructure has expanded faster than institutional capacity, AI adoption strategies should therefore prioritize responsible AI governance, inclusive skills development, workforce transition mechanisms, and equitable access to digital opportunities. A productivity-oriented AI strategy should not focus solely on technology deployment but also on ensuring that benefits are broadly distributed across sectors and population groups.

Because AI’s productivity dividend is mediated by workforce human capital, the practical implication of the preceding findings is that Somalia’s highest-leverage near-term investment is not additional digital infrastructure but the alignment of higher-education and technical-vocational curricula with digital- and AI-relevant competencies, coupled with structured, ministry-coordinated digital-skills training for the existing public-sector workforce, which national surveys show is young and motivated but under-trained (Hassan et al., 2026). Absent this alignment, further infrastructure investment risks reproducing the pattern already visible in Somalia’s ICT and computer-science graduate cohorts, where formal technical training has expanded rapidly without a commensurate rise in employability or productive absorption into the labour market (Ahmed, 2023; Yasin Gelle et al., 2021).

The evidence therefore suggests that AI readiness in fragile states should not be interpreted as a linear technological progression. Instead, readiness represents a dynamic capability shaped by interactions among infrastructure, institutions, human capital, and labour-market structures. Somalia illustrates that countries may possess relatively advanced digital infrastructure while remaining constrained by governance and workforce limitations. Consequently, AI strategies should prioritize capability formation and institutional alignment rather than technology acquisition alone.

## 6 Theoretical implications

This review contributes to the theoretical understanding of AI readiness in fragile-state contexts in three important ways. First, it extends the application of the Technology–Organization–Environment (TOE) framework beyond conventional organizational adoption settings by demonstrating that AI readiness in fragile states is a multidimensional capability shaped by the interaction between technological resources, institutional capacity, governance structures, and external ecosystem conditions. While traditional AI readiness models often emphasize infrastructure availability and organizational preparedness, the Somali case demonstrates that the presence of digital infrastructure alone does not guarantee meaningful AI adoption when institutional coordination, regulatory capacity, and workforce capabilities remain limited.

Second, the review advances the role of human capital theory in explaining the productivity mechanism underlying AI adoption. Rather than conceptualizing AI readiness as a direct predictor of workforce productivity, this study proposes a mediated relationship in which technological, organizational, and environmental readiness strengthen AI adoption capability, which subsequently enables effective human–AI collaboration, workforce augmentation, and productivity improvement. This perspective highlights that the value generated by AI depends not only on access to technology but also on the skills, adaptability, and absorptive capacity of individuals and organizations. Third, this review suggests that AI readiness frameworks developed primarily in high-income or institutionally stable contexts require adaptation when applied to fragile and conflict-affected states. In such settings, readiness pathways may not follow a linear progression from infrastructure development to institutional strengthening and AI adoption. Somalia illustrates an alternative pathway in which privately developed digital infrastructure has expanded faster than formal governance and human-capital systems. Therefore, theoretical models of AI readiness in fragile states should incorporate contextual factors including state capacity, private-sector digital ecosystems, donor influence, labour-market constraints, and institutional resilience.

Collectively, these theoretical insights suggest that AI readiness should be understood as an evolving capability-building process rather than a static technological condition. The integration of TOE and human capital theory provides a more context-sensitive framework for explaining how fragile states can transition from digital connectivity toward productive and inclusive AI adoption.

## 7 A proposed conceptual and policy framework for bridging the AI readiness gap in Somalia

### 7.1 Framework rationale

Building on the evidence synthesized from the systematic review and extending this evidence through context-specific policy interpretation, this study proposes a conceptual and policy-oriented AI Readiness Framework for Somalia (Figure 3). The framework is not presented as a validated predictive model or implementation blueprint; rather, it represents an evidence-informed analytical model derived from systematic synthesis and theoretical integration. Its purpose is to organize existing evidence, identify priority intervention domains, and generate testable propositions for future empirical validation. The four pillars reflect recurring determinants identified across the reviewed literature, while the prioritization, sequencing, and stakeholder roles represent an analytical extension developed by the authors to translate evidence into a context-specific policy framework. The four pillars extend the TOE framework by operationalizing human capital readiness as a distinct intervention domain, reflecting the central role of workforce capability in fragile-state AI adoption. The mapping process involved linking each pillar to specific themes identified during synthesis, identifying supporting evidence sources, assessing contextual relevance, and distinguishing evidence-supported recommendations from author-developed strategic proposals.

**Figure 3:**
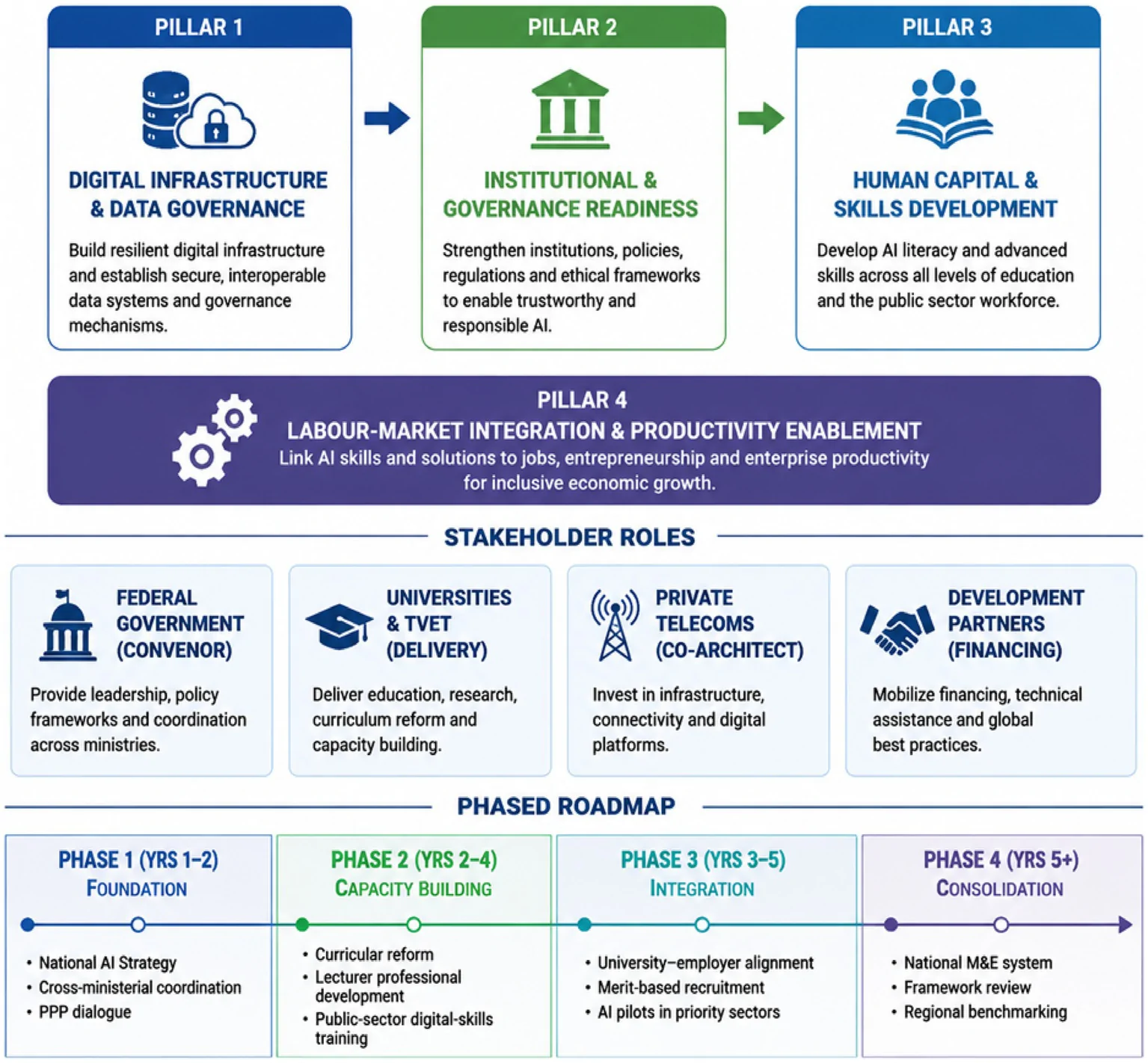
Proposed conceptual AI readiness framework for Somalia.

### 7.2 The four pillars

#### 7.2.1 Pillar 1: digital infrastructure and data governance

Rather than building new physical infrastructure from a low base, this pillar focuses on consolidating and governing what already exists. The evidence suggests that priority areas may include establishing a national data-governance framework that defines standards for interoperability between government systems and the mobile-money/telecommunications infrastructure that already carries the bulk of Somalia’s digital transactions, building on the demonstrated productivity and risk-sharing benefits mobile-money infrastructure has delivered elsewhere in East Africa (Chonka et al., 2025; Jack and Suri, 2014); negotiating formal public–private data-sharing and interoperability agreements with the dominant telecommunications conglomerates; and extending affordable connectivity specifically to underserved regions and to public universities and technical-vocational institutions, where infrastructure gaps most directly constrain digital-skills training (Hassan et al., 2026). Because digital financial inclusion elsewhere in Africa has been shown to depend as much on digital literacy as on infrastructure access (Elouaourti and Ibourk, 2024; Adel, 2024), infrastructure consolidation should be paired from the outset with parallel financial- and digital-literacy programming rather than treated as a purely technical undertaking.

#### 7.2.2 Pillar 2: institutional and governance readiness

This pillar addresses the organizational/environmental deficit identified as the most binding constraint in Section 5. Priority actions include developing Somalia’s first national AI and digital transformation strategy, explicitly co-designed with the private telecommunications sector rather than assuming public-sector primacy; embedding digital-skills and AI-literacy requirements into the existing public-sector ICT training initiatives that surveys show are welcomed by civil servants but currently uncoordinated (Osuma, 2025); and establishing a cross-ministerial digital governance coordination body to prevent the fragmentation that currently characterizes ICT policy across education, communications, and public-service ministries.

#### 7.2.3 Pillar 3: human capital and skills development

This pillar directly targets the mechanism identified as the highest-leverage intervention point, consistent with human capital theory’s long-standing account of education and training as productivity-enhancing investment (Zhai et al., 2025). Priority actions include reforming higher-education and technical-vocational curricula to embed digital literacy, data literacy, and foundational AI competencies, consistent with the curricular-reform recommendations emerging from the wider Sub-Saharan African evidence (Ndibalema, 2025; Akongo et al., 2025); investing in structured professional development for university lecturers and TVET instructors, who are repeatedly identified as under-prepared to teach digital content (Ndibalema, 2025); institutionalizing formal linkage mechanisms between TVET providers and employers, of the kind shown to reduce skills mismatch and unemployment elsewhere in the Horn of Africa (Kebede et al., 2024); and expanding applied, employer-linked training pathways that directly address the practical-skills and English-language-proficiency gaps identified as drivers of graduate unemployment (Ahmed, 2023; Yasin Gelle et al., 2021), while supporting parallel, platform-based digital-skills and entrepreneurship training of the kind shown to improve youth employability elsewhere on the continent (Neema Mariette et al., 2025). Comparative evidence from digital capacity-building in African health institutions further indicates that any such training should be structured, sustained, and institutionally embedded rather than one-off, since positive attitudes toward digital tools do not reliably translate into effective use without facilitating organizational conditions (Kiragga et al., 2025; El Arab et al., 2025).

#### 7.2.4 Pillar 4: labour-market integration and productivity enablement

This pillar closes the loop between skills development and productive employment. Priority actions include establishing structured university–employer curriculum-alignment mechanisms, of the kind piloted by international labour-mobility programs in Somalia, to ensure that graduate output tracks actual labour-market demand rather than credential supply alone; introducing merit-based recruitment safeguards in public and formal-sector hiring to ensure that skills investments translate into productivity gains rather than being neutralized by non-meritocratic allocation (Ahmed, 2023); and piloting sector-specific AI-augmentation initiatives in Somalia’s most digitally advanced sectors, mobile financial services and telecommunications, where existing infrastructure and private-sector capability could support early, demonstrable productivity gains.

### 7.3 Phased implementation roadmap

See Table 2

**Table 2:** Indicative phased implementation roadmap for the proposed conceptual AI readiness framework for Somalia.

Phase	Timeframe (indicative)	Principal actions
Phase 1: Foundation	Years 1–2	National digital/AI strategy development; cross-ministerial coordination body established; public–private data-governance dialogue initiated with telecommunications conglomerates
Phase 2: Capacity Building	Years 2–4	Curricular reform in higher education and TVET; lecturer/instructor professional development; structured, coordinated public-sector digital-skills training rollout
Phase 3: Integration	Years 3–5	University–employer alignment mechanisms operationalized; merit-based recruitment safeguards introduced; sector-specific AI-augmentation pilots launched in telecommunications and financial services
Phase 4: Consolidation	Years 5+	National AI readiness monitoring and evaluation system established; framework reviewed and adapted based on pilot outcomes and evolving regional AI readiness benchmarks

### 7.4 Stakeholder roles

The framework assigns differentiated but interdependent roles. The Federal Government of Somalia and relevant Federal Member State ministries are positioned as the convening and regulatory actor, responsible for strategy development, cross-ministerial coordination, and public-sector capacity building. Somali universities and TVET institutions are positioned as the primary delivery mechanism, responsible for curricular reform and instructor development. The private telecommunications and mobile-money sector, given that it demonstrated an infrastructural and quasi-governmental role (Chonka et al., 2025), is positioned as an essential co-architect rather than a peripheral stakeholder. Development partners and international organizations are positioned as technical-assistance and financing partners, particularly for strategy development and capacity building, consistent with the broader finding that capacity development in fragile and conflict-affected states is most effective when tailored to a country’s specific level of fragility and absorptive capacity rather than transplanted from stable-state models.

The Somali diaspora, estimated at over 2 million individuals, represents a distinctive and potentially transformative stakeholder in the AI readiness landscape. The diaspora has been central to Somalia’s telecommunications and mobile-money infrastructure, with diaspora-owned telecommunications conglomerates driving the sector’s expansion (Chonka et al., 2025). The diaspora also constitutes a substantial reservoir of professional human capital, including individuals with advanced technical and AI-relevant expertise gained in higher-income countries. Diaspora engagement mechanisms should include: (a) structured diaspora professional networks for university curriculum development and guest lectures; (b) diaspora investment vehicles for digital infrastructure and EdTech ventures; (c) knowledge-exchange programs linking Somali professionals abroad with public-sector institutions; and (d) diaspora advisory panels for national AI strategy development. These mechanisms are consistent with the cross-country evidence that diaspora engagement functions as a distinct channel through which low-income fragile states can convert external human and financial capital into domestic institutional capacity (Abbas, 2026). The diaspora should be positioned as a cross-cutting enabler across all four pillars rather than confined to a single pillar.

## 8 Future research

Because the proposed conceptual AI readiness framework was derived from systematic evidence synthesis rather than primary empirical testing, it should be considered a theory-informed conceptual model requiring empirical validation. Future research should apply Delphi methodology, expert consultation, stakeholder interviews, and pilot implementation studies involving policymakers, universities, private technology companies, and development partners to assess its feasibility, relevance, and measurable impact. This review identifies five priorities for future research. First, primary empirical studies, survey-based, qualitative, or mixed-methods, are urgently needed to directly assess AI readiness and awareness among Somali public institutions, universities, and private-sector employers, given the near-absence of AI-specific Somalia data identified in this review. Such studies should examine not only awareness and attitudes but also actual AI adoption patterns, barriers to use, and specific skills gaps that employers identify as most constraining. Second, longitudinal research tracking the labour-market outcomes of Somalia’s rapidly expanding computer-science and ICT graduate cohorts is essential to test whether curricular reform of the kind proposed in Pillar 3 improves employability and productivity outcomes. This would require establishing baseline data on graduate employment rates, skills assessments, and employer satisfaction. Third, comparative research examining how other fragile, telecommunications-advanced but institutionally weak states have sequenced AI readiness investment would help validate or refine the sequencing logic proposed in this framework. Fourth, implementation research evaluating specific interventions, public-private partnership models for AI governance, digital-skills curriculum reforms, and diaspora engagement mechanisms, would generate actionable evidence to inform policy and practice through collabouration between researchers, policymakers, and practitioners.

## 9 Conclusion

This systematic review synthesized peer-reviewed evidence on AI readiness, digital skills, and workforce capability in Somalia and comparable fragile, low-connectivity Sub-Saharan African contexts. The findings indicate that Somalia’s AI readiness gap is driven less by digital infrastructure limitations and more by institutional, governance, data ecosystem, and human-capital constraints. Although privately developed telecommunications and mobile-money systems have created important digital foundations, these assets have not yet been matched by coordinated AI governance mechanisms, workforce capabilities, or labour-market integration pathways. Theoretically, this review contributes to the AI readiness literature by demonstrating that readiness in fragile states should be understood as a multidimensional and sequential capability rather than a technology-access condition alone. By integrating the Technology–Organization–Environment framework with human capital theory, the study proposes that technological, organizational, and environmental readiness influence workforce outcomes through intermediate mechanisms of AI adoption capability, organizational absorptive capacity, and effective human–AI integration.

From a policy perspective, the proposed conceptual AI readiness framework provides an evidence-informed structure for organizing policy priorities and future research. Importantly, this framework should not be interpreted as a validated model of AI readiness outcomes. Rather, it represents a theory-informed policy framework generated through systematic evidence synthesis that requires validation through expert consensus, stakeholder engagement, empirical readiness assessment, and longitudinal implementation studies. To our knowledge, this review represents the first Somalia-specific conceptualization of AI readiness derived from available peer-reviewed evidence. However, because it was generated through secondary evidence synthesis, its dimensions, relationships, and implementation sequencing remain hypotheses requiring empirical testing. Future validation should involve Delphi consensus studies with AI experts, policymakers, educators, private-sector stakeholders, and development partners, followed by quantitative readiness assessments and pilot implementation studies.

Future research should prioritize empirical assessment of AI readiness among Somali institutions, universities, and employers; evaluation of digital-skills interventions; and longitudinal studies examining whether improvements in AI capability translate into measurable workforce and organizational outcomes. More broadly, the Somali case demonstrates that successful AI transformation in fragile states depends not only on technological availability but on the alignment of digital infrastructure, institutional capacity, skilled human resources, and inclusive governance mechanisms.

## Data Availability

The data extraction form, quality appraisal records, and search strategies are available from the corresponding author upon reasonable request.
