# Supplementary material for: Artificial intelligence readiness in fragile states: a systematic review and theory-informed framework for workforce transformation in Somalia
Source: Front Artif Intell. 2026 Oct 16;9:1928521. doi: 10.3389/frai.2026.1928521 (PMC13719570; doi:10.3389/frai.2026.1928521)
Supplement: Supplementary file 1 [file Supplementary_file_1.docx]

**Supplementary Table 1.** *Quality Appraisal of Included Studies*

| **No.** | **Study** | **Study Design** | **Assessment Approach** | **Quality Rating** | **Confidence Level** | **Influence on Synthesis** |
| --- | --- | --- | --- | --- | --- | --- |
| 1 | Hirtranusi et al. (2026) | Systematic Literature Review | JBI Critical Appraisal Checklist for Systematic Reviews | High | High confidence | Provided primary evidence on AI readiness dimensions and TOE-based synthesis |
| 2 | Demaidi (2023) | Conceptual/policy analysis | Critical narrative appraisal of conceptual contribution and policy relevance | Moderate | Moderate confidence | Used to contextualize AI strategy development in developing countries |
| 3 | Mienye et al. (2024) | Structured literature review | JBI Review Appraisal Checklist | High | High confidence | Provided evidence on AI adoption barriers in Africa |
| 4 | Aderibigbe et al. (2023) | Narrative/conceptual review | Critical narrative appraisal | Moderate | Moderate confidence | Supported interpretation of AI implementation barriers in developing countries |
| 5 | Chonka et al. (2025) | Qualitative field study | CASP Qualitative Checklist | High | High confidence | Provided Somalia-specific evidence on digital governance and private-sector infrastructure |
| 6 | Adams et al. (2024) | Global index/report analysis | AACODS Checklist | High | High confidence | Used as contextual benchmarking evidence for AI governance readiness |
| 7 | Ahmed (2023) | Cross-sectional survey | JBI Analytical Cross-sectional Checklist | Moderate | Moderate confidence | Supported evidence on graduate skills mismatch and employability challenges |
| 8 | Yasin Gelle et al. (2021) | Empirical case study | JBI Qualitative Checklist | Moderate | Moderate confidence | Supported understanding of labour-market constraints and graduate unemployment |
| 9 | Zhai et al. (2025) | Meta-analysis | AMSTAR-2 | High | High confidence | Supported human-capital and productivity mechanisms |
| 10 | Fan et al. (2025) | Firm-level empirical study | JBI Analytical Study Checklist | High | High confidence | Supported AI-productivity relationship mechanisms |
| 11 | Gao and Feng (2023) | Firm-level empirical study | JBI Analytical Study Checklist | High | High confidence | Supported AI-driven productivity pathways |
| 12 | Venkatesh et al. (2003) | Theory development study | Critical theoretical appraisal of conceptual contribution | High | High confidence | Supported technology adoption mechanisms through UTAUT |
| 13 | El-Jardali et al. (2023) | Scoping review | JBI Scoping Review Checklist | High | High confidence | Supported digital transformation challenges in fragile states |
| 14 | Page et al. (2021) | Reporting guideline/methodological paper | Methodological reference assessment (not quality rated) | Not applicable | Not applicable | Provided methodological guidance for PRISMA 2020 reporting and systematic review transparency |
| 15 | Arakpogun et al. (2021) | Conceptual analysis | Critical narrative appraisal | Moderate | Moderate confidence | Supported governance-related interpretation of AI adoption challenges |
| 16 | Ndibalema (2025) | Systematic review | JBI Systematic Review Checklist | High | High confidence | Supported digital literacy and higher-education findings |
| 17 | Akongo et al. (2025) | Systematic review | JBI Systematic Review Checklist | High | High confidence | Supported skills-development evidence |
| 18 | Kebede et al. (2024) | Literature review | JBI Review Checklist | Moderate | Moderate confidence | Supported TVET–industry linkage arguments |
| 19 | Neema Mariette et al. (2025) | Scoping review | JBI Scoping Review Checklist | High | High confidence | Supported digital skills and youth employability findings |
| 20 | Hassan et al. (2026) | Secondary data review | Critical narrative appraisal | Moderate | Moderate confidence | Supported Somalia higher-education digital transformation analysis |
| 21 | Uddin et al. (2026) | Cross-country empirical study | JBI Analytical Checklist | High | High confidence | Supported AI, employment, and innovation mechanisms |
| 22 | Yesuf et al. (2025) | Bibliometric and systematic review | JBI Review Checklist | High | High confidence | Supported organizational AI adoption factors |
| 23 | Jack and Suri (2014) | Panel empirical study | JBI Cohort Checklist | High | High confidence | Supported mobile-money productivity mechanisms |
| 24 | Elouaourti and Ibourk (2024) | Cross-country empirical study | JBI Analytical Checklist | High | High confidence | Supported digital financial inclusion mechanisms |
| 25 | Adel (2024) | Econometric study | JBI Analytical Checklist | High | High confidence | Supported digital literacy and technology adoption effects |
| 26 | Osuma (2025) | Secondary-data empirical study | JBI Analytical Checklist | Moderate | Moderate confidence | Supported digital finance evidence |
| 27 | Amare et al. (2024) | Cross-sectional survey | JBI Analytical Cross-sectional Checklist | Moderate | Moderate confidence | Supported evidence on digital competencies |
| 28 | Chereka et al. (2024) | Systematic review and meta-analysis | AMSTAR-2 | High | High confidence | Supported digital literacy determinants |
| 29 | Kiragga et al. (2025) | Multi-institution assessment | JBI Mixed-Methods Checklist | High | High confidence | Supported institutional capacity-building arguments |
| 30 | El Arab et al. (2025) | Systematic review | JBI Systematic Review Checklist | High | High confidence | Supported AI literacy and adoption readiness evidence |
| 31 | Abbas (2026) | Cross-country empirical study | JBI Analytical Checklist | High | High confidence | Supported diaspora engagement and institutional capacity arguments |
