# Supplementary material for: Artificial intelligence readiness in fragile states: a systematic review and theory-informed framework for workforce transformation in Somalia
Source: Front Artif Intell. 2026 Oct 16;9:1928521. doi: 10.3389/frai.2026.1928521 (PMC13719570; doi:10.3389/frai.2026.1928521)
Supplement: Supplementary file 2 [file Supplementary_file_2.docx]

**Supplementary Table 2.** *Characteristics of the studies included in the systematic synthesis.*

| **No.** | **Study** | **Year** | **Focus** | **Framework/Approach** | **Principal Findings/Contribution** |
| --- | --- | --- | --- | --- | --- |
| 1 | Hirtranusi et al. (2026) | 2026 | Organizational AI readiness (global) | PRISMA-based SLR; TOE framework | Consolidated 124 readiness indicators into 12 core themes; AI readiness is multidimensional and interdependent |
| 2 | Demaidi (2023) | 2023 | Artificial intelligence national strategy in a developing country | Conceptual/policy analysis | Developing countries face unique challenges in AI strategy formulation; global AI strategy frameworks often fail to account for local contexts |
| 3 | Mienye et al. (2024) | 2024 | AI and sustainable development in Africa | Structured literature review | Inadequate infrastructure, scarce data ecosystems, and skilled personnel shortages are principal barriers to AI adoption in Africa |
| 4 | Aderibigbe et al. (2023) | 2023 | AI in developing countries | Narrative/conceptual review | Infrastructure gaps, weak regulatory frameworks, and shortage of skilled personnel are key barriers to translating AI's potential into development outcomes |
| 5 | Chonka et al. (2025) | 2025 | Mobile money and state capacity in Somalia | Qualitative fieldwork/political economy | Private telecommunications conglomerates have assumed quasi-state functions; digital governance capacity concentrated outside formal state institutions |
| 6 | Adams et al. (2024) | 2024 | Global Index on Responsible AI 2024 (1st Edition) | Large-scale empirical index | Africa is the most heavily represented yet lowest-scoring region; South Africa leads the continent (27.61), while average African score is 5.83 out of 100 |
| 7 | Ahmed (2023) | 2023 | Graduate unemployment, Mogadishu | Empirical/survey-based | Skills mismatch is a leading structural driver of graduate unemployment; graduates are weak in practical, digital, and problem-solving competencies |
| 8 | Yasin Gelle et al. (2021) | 2021 | Graduate unemployment, Somalia | Empirical/case study | Skills mismatch and weak career guidance are leading drivers of graduate unemployment, compounded by non-meritocratic recruitment practices |
| 9 | Zhai et al. (2025) | 2025 | Country-level human capital and organizational performance | Meta-analysis | Country-level human capital moderates the relationship between high-performance work systems and organizational performance |
| 10 | Fan et al. (2025) | 2025 | AI, human capital, and firm total factor productivity | Firm-level empirical (China) | AI technology raises total factor productivity by increasing the proportion of highly skilled labour and generating capital-skill complementarities |
| 11 | Gao & Feng (2023) | 2023 | AI and firm productivity | Firm-level empirical (China) | AI penetration increases firm productivity through value-added, skill-biased, and technology-upgrading effects |
| 12 | Venkatesh et al. (2003) | 2003 | Technology acceptance and use | UTAUT model development | Performance expectancy, effort expectancy, social influence, and facilitating conditions determine technology adoption |
| 13 | El-Jardali et al. (2023) | 2023 | Digital health in fragile MENA states | Scoping review | Digital tools show promise in fragile states, but success depends on multi-stakeholder governance partnerships and context-specific evaluation |
| 14 | Page et al. (2021) | 2021 | Systematic review methodology | PRISMA 2020 guideline | PRISMA 2020 provides a transparent, replicable structure for reporting systematic reviews |
| 15 | Arakpogun et al. (2021) | 2021 | AI adoption across Africa | Conceptual analysis, Global North/South comparison | Global South countries must first address governance deficits and institutional capacity gaps before AI can flourish |
| 16 | Ndibalema (2025) | 2025 | Digital literacy gaps, Sub-Saharan Africa HEIs | Systematic review | 21st-century digital skills remain insufficiently developed due to inadequate digital infrastructure, underprepared lecturers, and outdated ICT policy |
| 17 | Akongo et al. (2025) | 2025 | 21st-century skills education, Sub-Saharan Africa | Systematic review | Life-skills and soft-skills integration into education systems remains inconsistent, constrained by infrastructural gaps and teacher preparedness |
| 18 | Kebede et al. (2024) | 2024 | TVET–industry linkage and employability, Ethiopia | Literature review | Weak linkage between vocational training and industry is a central, remediable cause of skills mismatch |
| 19 | Neema Mariette et al. (2025) | 2025 | Digital technology and youth unemployment, Africa | Scoping review | Digital-skills development enhances youth employability; digital platforms foster entrepreneurship by expanding access to markets |
| 20 | Hassan (2026) | 2026 | ICT and digital learning readiness, Somali higher education | Secondary data/policy review | Digital learning readiness highly uneven across institutions; shaped by disparities in connectivity, affordable data, computing devices, and institutional support |
| 21 | Uddin et al. (2026) | 2026 | AI, technological innovation, and employment | Cross-country empirical | AI and technological innovation reshape employment structures; outcomes depend on pre-existing digital and human-capital readiness |
| 22 | Yesuf et al. (2025) | 2025 | AI adoption in SMEs | Bibliometric and systematic review | Organizational readiness, digital infrastructure, and human capital jointly determine whether firms capture AI's benefits |
| 23 | Jack & Suri (2014) | 2014 | Mobile money and household risk-sharing, Kenya | Panel empirical study | Mobile money materially improved households' ability to share risk and smooth consumption after economic shocks |
| 24 | Elouaourti & Ibourk (2024) | 2024 | Digital financial inclusion drivers, Africa | Cross-country empirical (PCA index) | Digital financial inclusion shaped jointly by infrastructure access and digital literacy; women and less-educated populations face disproportionate exclusion |
| 25 | Adel (2024) | 2024 | Digital literacy and financial inclusion, emerging economies | Cross-regional GMM empirical | Digital literacy and technology adoption jointly determine the extent to which digital financial services translate into genuine financial inclusion |
| 26 | Osuma (2025) | 2025 | Digital financial services and economic inclusion, Sub-Saharan Africa | Empirical (secondary data) | Digital financial services adoption linked directly to measurable gains in economic inclusion |
| 27 | Amare et al. (2024) | 2024 | Digital competencies of health educators, Ethiopia | Cross-sectional survey | Only a small minority of health educators reached advanced digital-skill levels; the majority clustered at beginner or exploratory stages |
| 28 | Chereka et al. (2024) | 2024 | Digital literacy of health professionals, Ethiopia | Systematic review and meta-analysis | Highly variable digital-literacy levels strongly associated with prior training exposure and institutional support |
| 29 | Kiragga et al. (2025) | 2025 | Data-science capacity, African health institutions | Multi-institution capacity assessment | Data governance, infrastructure, and workforce training capacity varied substantially between otherwise comparable institutions |
| 30 | El Arab et al. (2025) | 2025 | AI readiness among nurses/nursing students | Systematic review | Attitudes and self-reported readiness consistently outpaced actual AI literacy; facilitating conditions determine whether positive attitudes translate into effective use |
| 31 | Abbas (2026) | 2026 | Technology, institutions, migration, and state fragility | Cross-country GMM empirical | Diaspora engagement functions as a distinct channel for converting external human and financial capital into domestic institutional capacity in fragile states |
